# Walk on the wild side: a response to Zaina

**DOI:** 10.1186/s43682-023-00019-z

**Published:** 2023-05-17

**Authors:** Luca Chiapperino, Francesco Paneni

**Affiliations:** 1 STS Lab, Institute of Social Sciences, Faculty of Social and Political Sciences, University of Lausanne, Quartier UNIL-Mouline, Bâtiment Géopolis, Bureau 5556, CH-1015 Lausanne, Switzerland; 2 Center for Translational and Experimental Cardiology (CTEC), University Hospital Zurich, University of Zurich, CH-8091 Zurich, Switzerland; 3 Department of Cardiology, University Heart Center, University Hospital Zurich, Zurich, Switzerland

We appreciated Silvio Zaina’s comment on our paper *Why epigenetics is (not) a biosocial science and why that matters.* We thank him for acknowledging that dialogue around the biosocial aspects of epigenetics is worth the efforts of the community. In turn, we consider commentaries like Zaina’s an integral part of such a debate: without a critical and reflective approach to interdisciplinarity, the analysis of the social and epistemological dimensions of epigenetics risks remaining highly programmatic and abstract. At the same time, we would like to take the opportunity of this reply to express some doubts about his comment.

In his reply, the author pins evidence of the genetic basis of epigenetic differences (mostly in methylation [1]) against our proposal for a deeper engagement with the biosocial dimensions of epigenetics. If the individual characteristics of the epigenome are “for the vast majority” ([2] p.3) dependent on interindividual genetic differences, should the field embrace a better understanding of the social and/or environmental ramifications of these differences? Zaina provides a negative answer to this question, based on two reasons. One is that he believes there is little to be learnt from studies of the environmentally driven variation in epigenetic predispositions to disease: the “grip” ([2] p.3) of genetics over epigenetics is so that the latter should be renamed “paragenetics” ([2] p.4). The second is that by following our call towards a complexification of the biosocial tools of epigenetics, one runs the risk of reifying social differences under study in epigenetics as the genetic differences that have a “grip” on them. We will address these two points in turn.

Zaina’s reading of the relationship between genetics and epigenetics contains several elements of truth. Although often overlooked, these can be found as well in a thread of well-known philosophical, historical, and sociological work [3–5]: epigenetic differences may not *just* be the marks, the product, or the biological correlates of one’s unique exposures to the (ecological, material, and social) environment(s). The epigenome is the result of developmental trajectories (i.e., timing) and individual genetic differences too (i.e., variation). We are aware of this important nuance and never intended to suggest otherwise. But—and this will be our focus here—one should be equally careful to avoid drawing the wrong conclusion from this affirmation. Claiming from the presence of “genetics’ grip on the epigenome” ([2] p.3) that (i) environmental determinants are not important, or that (ii) the epigenome is in “essence” under the control of the genome (p.4), is as problematic as the claim of an epi-genome just at the mercy of its (ecological, material, and social) environment(s). The reason is that *this is not an either/or matter*. As shown extensively by scholarship on developmental epigenetics [6, 7] or the nature-nurture debate [8], this dichotomous way of framing the issue is the problem. Genetic and non-genetic factors constitute “a dynamic, mutually dependent, relational developmental system” within which different “components” and levels of organization “coact to promote the emergence, maintenance, or modification of phenotypic traits” [6]. Our point builds on the need to transcend any dichotomous view of genes and environments in developmental relations and is at its core a pragmatic call towards methodological symmetry: why is the complexity of methods, tools, and questions studying biological variables of developmental systems unmatched when it comes to socio-environmental variables and their biological ramifications?

This leads us to the second point raised by Zaina’s comment: the risk of reifying, through epigenetics, social differences into the (alleged) 'real' genetic differences hidden behind the epigenome. We do not believe the complexity of such phenotypes and human conditions will ever be amenable to genetic differences, let alone be “bound to” be reframed as “genetics-related phenomena” ([2] p.5). Not because it is impossible to misinterpret genetic and/or epigenetic differences as privileged informational sources in the face of their deep social, historical, and environmental ramifications: critical scholarship raising the concern about risky revivals of sociobiological ideas in epigenetics has repeatedly hammered this nail on its head [9–13]. Rather, because from our perspective it is impossible to reduce complex phenotypic differences to *either* one of its components (i.e., the genome and the environment) [8]. There might be specific phenotypes for which a genetic difference may be *more instructive* to understand the roles and contributions of a given epigenetic difference relating (among other things) to environmental exposures or developmental trajectories (cf. [14]). Yet, even these phenotypes would have to be explained as the product of the biosocial components of the system (i.e., the organism). Once one recognizes this crucial distinction, one can see how we do not share Zaina’s caution about the risk of affirming the primacy of the genome through the epigenome. There exists a risk of misinterpreting the biosocial dimensions of the epigenome. Yet, we ask: is this risk enough of a reason to give up on the “noble and useful enterprise” to reduce the “daylight between epigenetics and fine-grained mapping of the social milieu” ([2] p.5)?

Experimenting with the epistemic foundations and political undertones of a biosocial epigenetics is no easy task. However, we believe there exist few alternatives to walking into this territory—we owe the peregrination metaphor to Zaina—without taking the (controlled and relative) risk of thinking and writing about these issues with colleagues from different trajectories, if not worldviews. This may be far from someone’s comfort zone of pipettes and tubes, but it may be nonetheless a productive and exciting stroll. Scientists, take a walk on the wild side.

## Data Availability

Data sharing not applicable to this article as no datasets were exclusively generated for the current study.
